# Hospital Meetings

**Published:** 1896-07-04

**Authors:** 


### HOSPITAL MEETINGS.

BRITISH HOME FOR INCURABLES, STREATHAM.

After a stormy morning a beautifully fine and sunny afternoon favoured the garden party held on Tuesday at the British Home for Incurables, and helped to make the entertainment an unqualified success. The Duchess of Sutherland had promised to open a sale of the inmates' work, of which the proceeds would be devoted to their own benefit, but was unavoidably prevented from coming, and the little ceremony was performed by Mrs. Tritton. The crowd round the stall seemed to point to brisk buying during the afternoon. A variety of entertainments were provided for the amusement of the guests, who, it is hoped, came with well-lined pockets and open hands. The Duke of Newcastle gave an exhibition of the Röntgen Rays, which proved as popular as usual, and an "Illusionist Entertainment" collected a large audience. The band of the Seaforth Highlanders played on the lawn. The home is a charming building, an ideal spot for invalids. It stands very high, almost every window commanding good views over Norwood and Streatham. The wide corridors, floored like the rooms with unpolished wood blocks, have sunny windows at unexpected corners, with low-cushioned seats; the rooms, some for four or six beds, some single, are bright and cheerful, and most comfortably furnished. The invalids have plenty of wheel chairs and sofas, so that those who are able can be taken out on the sunny terrace, or into the window embrasures, with a minimum of fatigue. The day rooms on each floor are particularly pleasant apartments. The chapel at one end of the building is especially arranged for the invalid chairs and couches to be taken in easily, and a gallery enables the patients on the first floor to join in the services without going downstairs. The contented looks of the invalids bore testimony to the good care of Mrs. Taylor, the matron, and Nurse Groome, the trained head nurse.

